# Associations of intrinsic capacity, fall risk and frailty in old inpatients

**DOI:** 10.3389/fpubh.2023.1177812

**Published:** 2023-10-10

**Authors:** Shanshan Shen, Yanhong Xie, Xingkun Zeng, Lingyan Chen, Huilan Guan, Yinghong Yang, Xiushao Wu, Xujiao Chen

**Affiliations:** Department of Geriatrics, Zhejiang Hospital, Hangzhou, China

**Keywords:** older adults, hospitalized, intrinsic capacity, fall, frailty

## Abstract

**Introduction:**

This study explored the associations of intrinsic capacity (IC), fall risk, and frailty in geriatric inpatients.

**Methods:**

A total of 703 hospitalized patients aged 75 years or older were recruited for this retrospective observational study from Zhejiang Hospital using a comprehensive geriatric assessment. The IC composite score was constructed from the scores of the Chinese version of the Mini-Mental State Examination, Short Physical Performance Battery, Short Form Mini Nutritional Assessment, 15-item Geriatric Depression Scale, and self-reported hearing and vision impairment. Adverse outcomes were recorded as the fall risk and frailty using the Morse Fall Scale and the Clinical Frailty Scale. Spearman’s correlation coefficient analyses and multivariate logistic regression models were used to explore the associations between IC, high fall risk, and frailty.

**Results:**

Declined IC composite scores were associated with increased risks of falls [odds ratio (OR) = 0.64, 95% confidence interval (CI): 0.57–0.72] and frailty (OR = 0.45, 95%CI: 0.37–0.54) among older hospitalized patients after adjusting for the related potential confounders. In addition, decreased cognitive, vitality, locomotion, and psychological scores were associated with increased adverse health conditions, with ORs ranging from 0.26 to 0.70. Vision impairment was observed to increase the risk of frailty (OR = 0.42, 95%CI: 0.23–0.76) after adjusting for the related potential confounders.

**Discussion:**

This study indicated that declined IC was associated with fall risk and frailty in older inpatients. Further prospective studies are needed to explore the longitudinal associations between baseline IC and subsequent risk of falls and frailty.

## 1. Introduction

Due to the rising population of older adults in China, the number of hospitalized older patients is increasing annually. Increased complicated conditions and functional declines brought by age and hospitalization, older patients aged ≥75 years old are a high-risk group for frailty and falls (1–4), and these adverse geriatric conditions can persist long after discharge (5–7). The long-term adverse effects of frailty and falls include subsequent loss of independence, disability, increased care needs, health expenditures, and mortality (8–12). Based on the reversibility of frailty and fall risk (13, 14), falls and frailty prevention during hospitalization may complement the in-hospital quality of medical and care goals of advancing disability and adverse events. Adopting person-centered interventions and care plans is critical for reducing adverse outcomes during and after hospitalization.

Intrinsic capacity (IC) is the core concept of functional ability proposed by the World Health Organization (WHO) to promote healthy aging (15). IC is defined as the physical and mental capacity of an individual, including cognition, locomotion, vitality, psychological, and sensory abilities (16). Previous studies have focused on community-level IC screening, assessment, and intervention among older adults (17, 18). Studies in community dwellings and nursing homes have revealed that IC validly predicts falls, frailty, disability, and mortality (19, 20). Impaired vitality, locomotion, and psychology domains have been reported to predict the incidence of future falls within two years, and cognitive decline was reported to be associated with an increased risk of activities of daily living dependence in the future (21). Another study from Singapore showed that a higher composite IC was associated with decreased risk of frailty progression, incident frailty, falls, health deterioration, and functional decline (22). However, few studies have integrated IC into rapid functional screening and assessment of older inpatients in general hospitals (23–25). A recent study investigating the association between IC and poor outcomes one year after discharge in an older hospitalized population found that higher IC composite scores at admission indicated a lower likelihood of disability and death (23). Despite effective tools and interventions for prevention, poor outcomes caused by recurrent falls and frailty progression remain common and expensive among older adults (10, 26). Indeed, the World Fall Guideline recommends that fall prevention and management can partly be achieved by improving IC domains, such as mobility, sensory function, cognitive function, and nutrition status (27). Consensus guidelines also emphasize that nutrition and exercise, which correspond to the two important components of IC nutrition and locomotion, are important intervention strategies for frailty prevention and management (28). Thus, it is important to explore the associations of in-hospital IC levels, fall risk, and frailty in older inpatients.

In addition, although the conceptual framework and dimensions of IC generally agree, the assessment methods of the five domains differ across studies, and no consensus has been reached on the best approach to compute a global composite score of IC to account for all dimensions holistically (29, 30). Considering the clinical utility of a summary score for routine geriatric assessment, Lopez-Ortiz et al. proposed an IC composite score that consisted of the characteristics of all five dimensions and weighted each dimension equally by reviewing the existing evidence (30). Based on the characteristics of vulnerability and variability among older inpatients, the proposed tools may avoid the risk of specific deficits compared to the ICOPE tools. Thus, we adopted an IC composite score to assess the holistic functional status of an individual and further explored the association between IC, fall risk, and frailty in geriatric inpatients.

## 2. Methods

### 2.1. Study design and participants

This retrospective observational study was based on the existing comprehensive geriatric assessment (CGA) database of older hospitalized patients in Zhejiang Hospital in China. The CGA database was established in 2014 to collect data on geriatric syndromes for older hospitalized patients. A total of 1624 potential patients were consecutively recruited between March 2014 and July 2022, and all data were obtained when the patients were in relatively stable condition. The inclusion criterion was older inpatients aged ≥75 years. The exclusion criteria were as follows: outpatients, inability to cooperate or refusal to complete the CGA, and incomplete data of importance such as IC, fall risk, and frailty. Ethical approval was obtained from the Medical Ethics Committee of Zhejiang Hospital (2013-25), and all patients provided written informed consent prior to data collection.

### 2.2. Data collection

Data were collected by trained professional nurses at the CGA through face-to-face interviews between March 2014 and July 2022. Demographic information including collection time, age, sex, educational level (coded as junior high school and below, higher school and above), marital status (categorized as married, unmarried, widowed, or divorced), religion, cigarette smoking, alcohol use, reason for admission, and medication use was collected. The body mass index was calculated using the height and weight measurements. Comorbidity was assessed using the Cumulative Illness Rating Scale for Geriatrics (CIRS-G). It includes 14 systemic disease categories, with severity in each category assessed on a scale of 0 to 4. Higher total CIRS-G scores indicate a higher comorbidity burden (31). Polypharmacy was determined as the concomitant use of ≥ five medications (32). The history of falls in the past year, fear of falling, walking aid use, dentures, and eating problems were also recorded. Pain was measured using the Numerical Rating Scale (NRS) (33), and an NRS score of ≥1 indicated the presence of pain. Urinary incontinence was considered when the score on the International Consultation on Incontinence Questionnaire-Short Form (ICIQ-SF) was 1 or higher (34).

### 2.3. IC assessment

Based on the WHO’s conceptual framework on IC and the existing literature, we integrated the five domains of cognition, locomotion, vitality, psychology, and sensation into a composite score (30). Each domain was scored a 0–2 range to stratify three statuses of functional impairment (0 = severely impaired; 1 = partially impaired; 2 = slightly impaired or fully preserved). The total IC composite score ranges from 0 to 10, with a higher composite score indicating a higher IC reserve.

Cognitive impairment was ascertained using the Chinese version of the Mini-Mental State Examination (35), and scores of 0–9, 10–26, and 27–30 points were computed as 0, 1, and 2, respectively. Locomotion was evaluated using the Short Physical Performance Battery (SPPB) (36, 37), which comprises a 4 m gait speed, five-repetition maximum chair rise, and static balance. SPPB scores of 0–2, 3–9, and 10–12 points were computed as 0, 1, and 2 points, respectively. The short form Mini Nutritional Assessment (MNA-SF) was used to assess vitality, with cut-off values of 0–7, 8–11, and 12–14 points (38). Depressive symptoms using the 15-item Geriatric Depression Scale (GDS-15) were used to determine psychological capacity (39). The cut-off GDS-15 scores used to classify depression severity were 5 and 10 (40). The sensory domain was assessed using self-reported hearing and vision impairment, which was categorized as total or severe loss (0 points), moderate loss (0.5 points), and normal or mild loss (1 point). Based on the available IC assessment database, vitality, and psychology domains were replaced with the MNA-SF and GDS-15 scale instead of the recommended assessment tools.

### 2.4. Fall risk assessment

Falls risk was assessed using the Morse Fall Scale (MFS) (41), which consists of six items: history of falling within three months, secondary diagnosis, ambulatory aid, intravenous or heparin lock, gait, and mental status. The total MFS score ranges from 0 to 145, with a score greater than 45 points indicating a high fall risk.

### 2.5. Frailty assessment

Frailty was evaluated using the Clinical Frailty Scale (CFS) (42), which has been validated in a Chinese hospital setting. The scale ranges from 1 (very fit) to 7 (severely frail), and a CFS score of ≥5 indicated frailty (43).

### 2.6. Statistical analysis

Data were analyzed using SPSS (version 26.0; SPSS, Chicago, IL, United States). The continuous variables were expressed as the mean ± standard deviation or median and interquartile range (IQR) as appropriate. Categorical variables are expressed as frequencies (N) and percentages (%). Differences in fall risk and frailty status among the baseline characteristics were assessed using the unpaired t-test, Mann-Whitney U-test, and chi-square test, where appropriate. Spearman’s correlation coefficient analyses were used to evaluate the associations between the IC composite, MFS, and CFS scores. Furthermore, multivariate logistic regression models to estimate odds ratios (ORs) and 95% confidence intervals (CIs) were used to explore the effect of IC on high fall risk and frailty, adjusted for potential variables in the univariate analysis. The potential variables with *P* < 0.05 in bivariate analysis and some critical variables reported by experts were selected in the multivariate logistic regression analysis. A *P*-value of <0.05 was considered statistically significant.

## 3. Results

**Figure 1:**
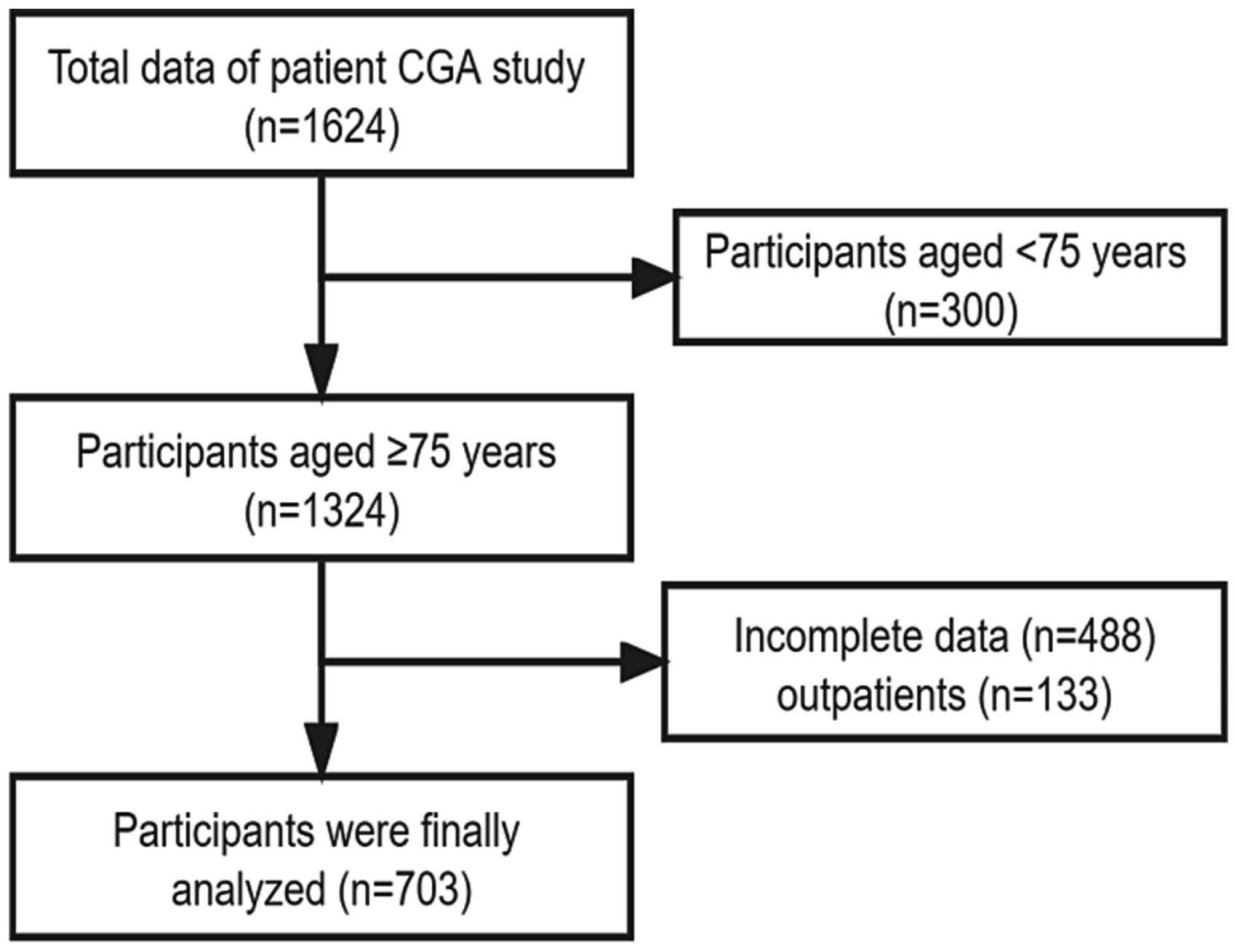
The flow chart of patients selection.

**Figure 2:**
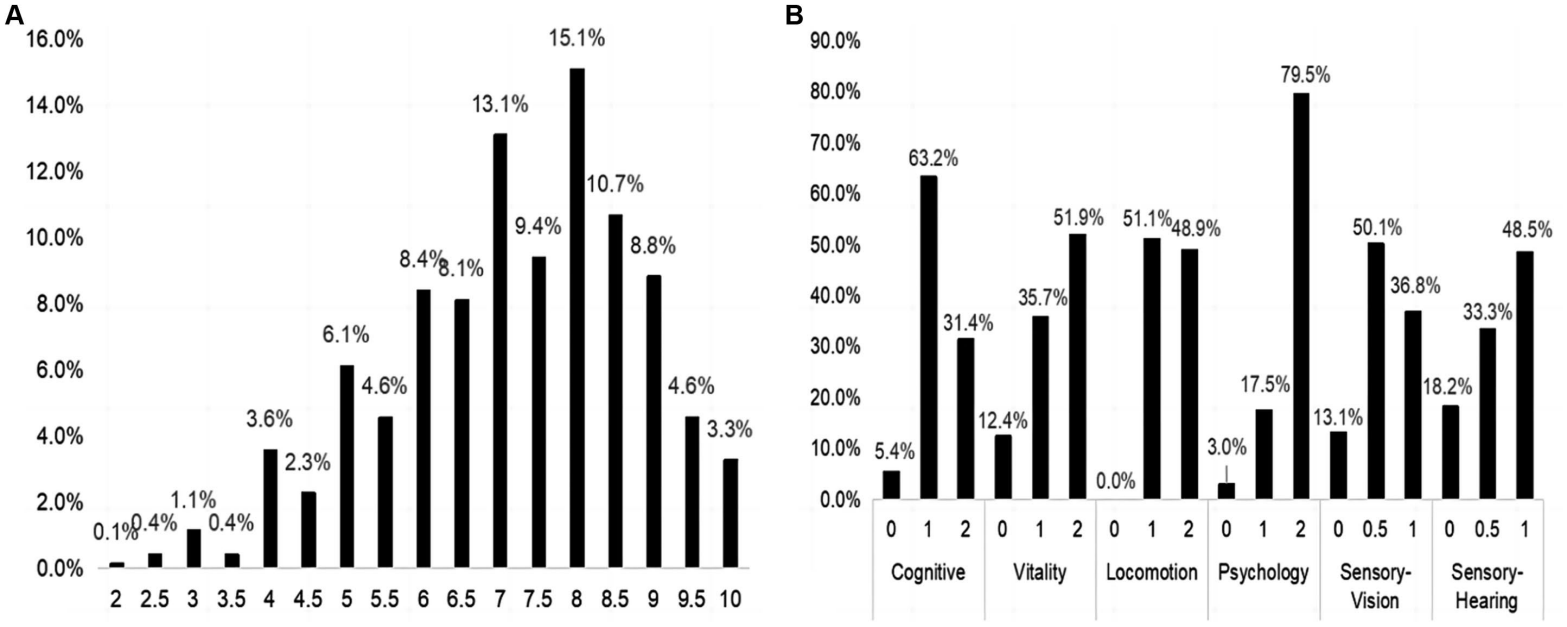
The distribution of IC composite score and its five domains. **(A)** The distribution of IC composite score in the total sample. **(B)** The distribution of IC domains in the total sample. IC, intrinsic capacity.

Figure 1 presents a flowchart of the patient selection. A total of 703 inpatients were included in the analysis. Among the included inpatients, 249,199 and 255 older inpatients were collected in the 2014–2016 year, 2017–2019 year, and 2020–2022 year, respectively. Among them, 57.3% were male, with a median age of 85 years, 66.1% were married, 55.3% had high school and above level of education. Figure 2A shows the distribution of the IC composite scores; the median IC composition score was 7.5 points (2–10 points). Additionally, the median IC composition score was 7.5, 7.5, and 7.0 points in the 2014–2016 year, 2017–2019 year, and 2020–2022 year, respectively. Regarding the IC domains, cognitive impairment (68.6%) was the most common, followed by sensory (vision 63.2%, hearing 51.5%), locomotion (51.1%), vitality (48.1%), and psychological impairments (20.5%), as shown in Figure 2B.

**Figure 3:**
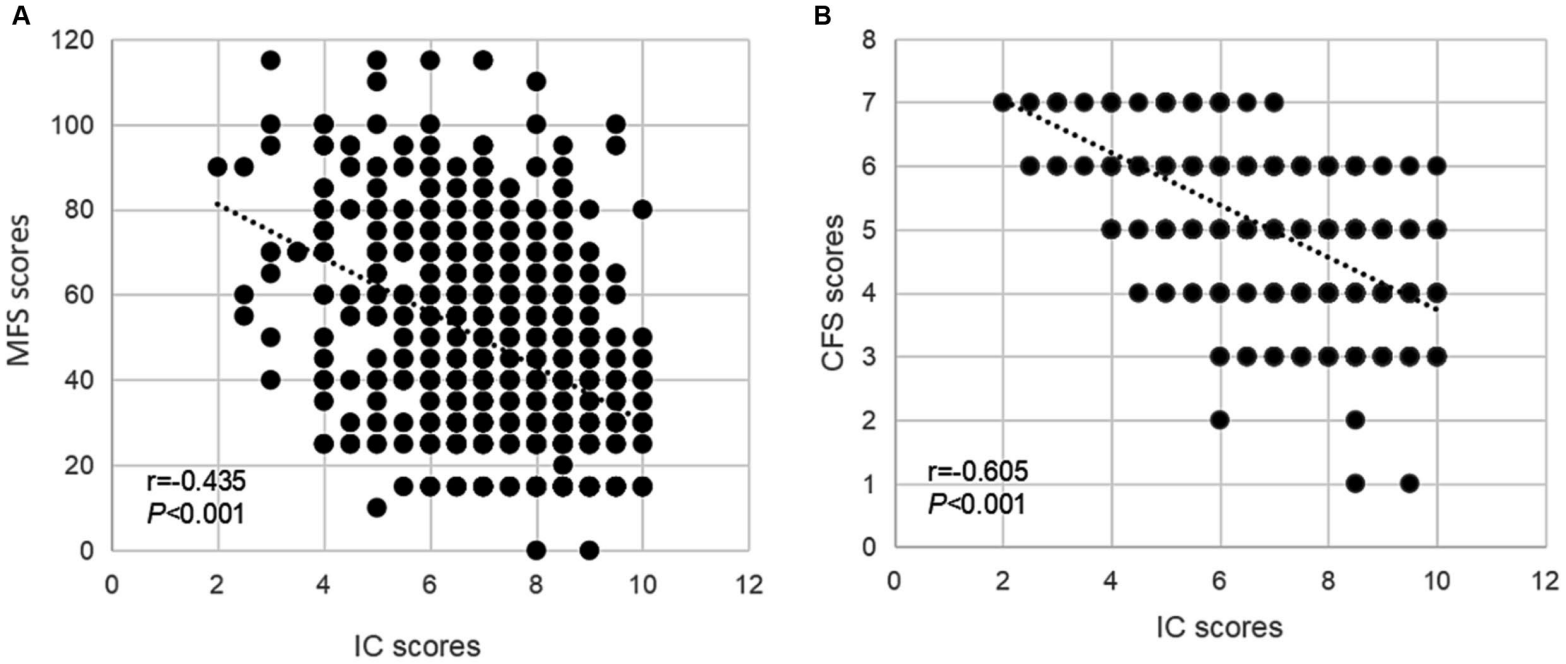
The associations of IC and geriatric health outcomes during the hospital stay. Spearman correlation coefficients were calculated between IC scores and **(A)** MFS scores, **(B)** CFS scores in older inpatients. IC, intrinsic capacity; MFS, Morse Fall Scale; CFS, Clinical Frailty Scale.

The general characteristics and sex-specified characteristics of the sample are listed in Table 1. Among the total inpatients, 327 (46.5%) exhibited a high fall risk, and 460 (65.4%) experienced frailty. Significant sex differences were observed in variables about age, marriage status, educational level, religious belief, smoking and drinking history, fear of falling, reason for admission, CIRS-G score, pain, and urinary incontinence (all P for trend <0.05), but not in IC composite score, fall risk, and frailty. As presented in Table 2, compared with inpatients without a high fall risk and frailty, inpatients who experienced these negative health conditions were older, had a higher percentage of fall history in the past year, had a fear of falling, used a walking aid, had eating problems, comorbidities, polypharmacy, urinary incontinence, and lower IC composite scores (all *P* < 0.05). As shown in Figure 3, the IC composite scores were negatively associated with the MFS and CFS scores (all *P* < 0.01). Furthermore, Table 3 shows that declined IC composite scores were associated with an increased risk of falls (OR = 0.64, 95%CI: 0.57–0.72) and frailty (OR = 0.45, 95%CI: 0.37–0.54) among older hospitalized patients after adjusting for the related potential confounders. Regarding the relationships between IC domains and negative health outcomes, multivariate regression models revealed that declined capacities in the cognitive, vitality, locomotion, and psychological domains were associated with increased adverse health conditions, and the ORs ranged from 0.26 to 0.70. However, vision impairment in the sensory domain was observed to increase the risk of frailty (OR = 0.42, 95%CI: 0.23–0.76) after adjusting for the related potential confounders.

**Table 1:** General characteristics and sex-specified characteristics of the total sample.

	Total sample (*n* = 703)	Males (*n* = 403)	Females (*n* = 200)	*P-*value
Collection time, n (%)				**0.003**
2014–2016	249 (35.4)	**164 (40.7)**	**85 (28.3)**	
2017–2019	199 (28.3)	**106 (26.3)**	**93 (31.0)**	
2020–2022	255 (36.3)	**133 (33.0)**	**122 (40.7)**	
Age, [median (IQR), years]	85 (81, 88)	**85 (81, 89)**	**84 (80, 87)**	**<0.001**
Married, n (%)	465 (66.1)	**297 (73.7)**	**168 (56.0)**	**<0.001**
High school or above, n (%)	389 (55.3)	**239 (59.3)**	**150 (50.0)**	**0.014**
Religion, n (%)	87 (12.4)	**28 (6.9)**	**59 (19.7)**	**<0.001**
Current or former smoker, n (%)	171 (24.3)	**162 (40.2)**	**9 (3.0)**	**<0.001**
Current or former drinker, n (%)	152 (21.6)	**131 (32.5)**	**21 (7.0)**	**<0.001**
BMI, [mean±SD, Kg/m^2^]	23.2 ± 3.7	23.3 ± 3.6	23.1 ± 3.7	0.386
Fall history in the past year, n (%)	170 (24.2)	100 (24.8)	70 (23.3)	0.650
Fear of falling, n (%)	409 (58.2)	**218 (54.1)**	**191 (63.7)**	**0.011**
Walking aids, n (%)	262 (37.3)	155 (38.5)	107 (35.7)	0.448
Dentures, n (%)	479 (68.1)	277 (68.7)	202 (67.3)	0.693
Eating problems, n (%)	316 (45.0)	173 (42.9)	143 (47.7)	0.212
Reason for admission, n (%)				**0.001**
Cardiovascular diseases	105 (14.9)	**64 (15.9)**	**41 (13.7)**	
Peripheral vascular diseases	332 (47.2)	**189 (46.9)**	**143 (47.7)**	
Nervous system diseases	70 (10.0)	**33 (8.2)**	**37 (12.3)**	
Respiratory diseases	56 (8.0)	**45 (11.2)**	**11 (3.7)**	
Other	140 (19.9)	**72 (17.9)**	**68 (22.7)**	
CIRS-G score, [(Median, IQR), scores]	9.0 (7.0, 9.0)	**10.0 (7.0, 13.0)**	**8.0 (6.0, 11.0)**	**0.001**
Polypharmacy (≥5 medications), n (%)	419 (59.6)	251 (62.3)	168 (56.0)	0.093
IC composite score, [median (IQR), scores]	7.5 (6.0, 8.5)	7.5 (6.5, 8.5)	7.3 (6.0, 8.5)	0.467
Pain, n (%)	308 (43.8)	**156 (38.7)**	**152 (50.7)**	**0.002**
Urinary incontinence, n (%)	272 (38.7)	**138 (34.2)**	**134 (44.7)**	**0.005**
High fall risk, n (%)	327 (46.5)	193 (47.9)	134 (44.7)	0.397
Frailty, n (%)	460 (65.4)	254 (63.0)	206 (68.7)	0.120

BMI, body mass index; CIRS-G, Cumulative Illness Rating Scale for Geriatrics, IC, intrinsic capacity. Significance difference *P* < 0.05 was shown in bold.

**Table 2:** Comparison of the general characteristics divided into the status of fall risk and frailty.

	High fall risk (*n* = 703)			Frailty (*n* = 703)		
	No (*n* = 376)	Yes (*n* = 327)	*P-*value	No (*n* = 243)	Yes (*n* = 460)	*P-*value
Collection time, n (%)			**<0.001**			**<0.001**
2014–2016	**151 (40.2)**	**98 (30.0)**		**98 (40.3)**	**151 (32.8)**	
2017–2019	**117 (31.1)**	**82 (25.1)**		**82 (33.7)**	**117 (25.4)**	
2020–2022	**108 (28.7)**	**147 (45.0)**		**63 (25.9)**	**192 (41.7)**	
Age, [median (IQR), years]	**84 (80, 87)**	**86 (83, 89)**	**<0.001**	**82 (78, 86)**	**86 (83, 89)**	**<0.001**
Male, n (%)	210 (55.9)	193 (59.0)	0.397	149 (61.3)	254 (55.2)	0.120
Married, n (%)	257 (68.4)	208 (63.6)	0.185	**185 (76.1)**	**280 (60.9)**	**<0.001**
High school or above, n (%)	219 (58.2)	170 (52.0)	0.096	**147 (60.5)**	**242 (52.6)**	**0.046**
Religion, n (%)	39 (10.4)	48 (14.7)	0.084	23 (9.5)	64 (13.9)	0.089
Current or former smoker, n (%)	**74 (19.7)**	**97 (29.7)**	**0.002**	60 (24.7)	111 (24.1)	0.869
Current or former drinker, n (%)	75 (19.9)	77 (23.5)	0.247	54 (22.2)	98 (21.3)	0.779
BMI [mean±SD, Kg/m^2^]	23.1 ± 3.4	23.3 ± 3.9	0.443	23.3 ± 3.1	23.2 ± 3.9	0.643
Fall history in the past year, n (%)	**38 (10.1)**	**132 (40.4)**	**<0.001**	**38 (15.6)**	**132 (28.7)**	**<0.001**
Fear of falling, n (%)	**200 (53.2)**	**209 (63.9)**	**0.004**	**110 (45.3)**	**299 (65.0)**	**<0.001**
Walking aids, n (%)	**66 (17.6)**	**196 (59.9)**	**<0.001**	**31 (12.8)**	**231 (50.2)**	**<0.001**
Dentures, n (%)	247 (65.7)	232 (70.9)	0.136	167 (68.7)	312 (67.8)	0.808
Eating problems, n (%)	**153 (40.7)**	**163 (49.8)**	**0.015**	**87 (35.8)**	**229 (49.8)**	**<0.001**
Reason for admission, n (%)			0.451			0.266
Cardiovascular diseases	60 (16.0)	45 (13.8)		43 (17.7)	62 (13.5)	
Peripheral vascular diseases	185 (49.2)	147 (45.0)		114 (46.9)	218 (47.4)	
Nervous system diseases	32 (8.5)	38 (11.6)		17 (7.0)	53 (11.5)	
Respiratory diseases	29 (7.7)	27 (8.3)		20 (8.2)	36 (7.8)	
Other	70 (18.6)	70 (21.4)		49 (20.2)	91 (19.8)	
CIRS-G score, [median (IQR), scores]	**8.0 (6.0, 11.8)**	**10.0 (7.0, 13.0)**	**0.001**	**8.0 (6.0, 11.0)**	**10.0 (7.0, 13.0)**	**<0.001**
Polypharmacy (≥5 medications), n (%)	**211 (56.1)**	**208 (63.6)**	**0.043**	**113 (46.5)**	**306 (66.5)**	**<0.001**
Pain, n (%)	**147 (39.1)**	**161 (49.2)**	**0.007**	95 (39.1)	213 (46.3)	0.067
Urinary incontinence, n (%)	**124 (33.0)**	**148 (45.3)**	**0.001**	**74 (30.5)**	**198 (43.0)**	**0.001**
IC composite score, [median (IQR), scores]	**8.0 (7.0, 8.5)**	**7.0 (5.5, 8.0)**	**<0.001**	**8.5 (7.5, 9.0)**	**7.0 (5.5, 8.0)**	**<0.001**
IC subdomains						
Cognition, [median (IQR), scores]	**1.0 (1.0, 2.0)**	**1.0 (1.0, 1.0)**	**<0.001**	**1.0 (1.0, 2.0)**	**1.0 (1.0, 1.0)**	**<0.001**
Vitality, [median (IQR), scores]	**2.0 (1.0, 2.0)**	**1.0 (1.0, 2.0)**	**<0.001**	**2.0 (1.0, 2.0)**	**1.0 (1.0, 2.0)**	**<0.001**
Locomotion, [median (IQR), scores]	**2.0 (1.0, 2.0)**	**1.0 (1.0, 2.0)**	**<0.001**	**2.0 (2.0, 2.0)**	**1.0 (1.0, 2.0)**	**<0.001**
Psychology, [median (IQR), scores]	**2.0 (2.0, 2.0)**	**2.0 (1.0, 2.0)**	**0.004**	**2.0 (2.0, 2.0)**	**2.0 (1.0, 2.0)**	**<0.001**
Vision, [median (IQR), scores]	**0.5 (0.5, 1.0)**	**0.5 (0.5, 1.0)**	0.300	**0.5 (0.5, 1.0)**	**0.5 (0.5, 1.0)**	**<0.001**
Hearing, [median (IQR), scores]	**0.5 (0.5, 1.0)**	**0.5 (0.5, 1.0)**	0.283	**1.0 (0.5, 1.0)**	**0.5 (0.5, 1.0)**	**0.004**

Significance difference *P* < 0.05 was shown in bold. BMI, body mass index; CIRS-G, Cumulative Illness Rating Scale for Geriatrics; IC, intrinsic capacity; IQR, inter-quartile range.

**Table 3:** Multivariate regression models of the associations of IC composition score and its subdomains, fall risk, and frailty.

	High fall risk^a^		Frailty^b^	
	OR (95%CI)	*P-*value	OR (95%CI)	*P-*value
IC composition score	**0.64 (0.57, 0.72)**	**<0.001**	**0.45 (0.37, 0.54)**	**<0.001**
IC subdomains				
Cognition	**0.38 (0.28, 0.53)**	**<0.001**	**0.33 (0.22, 0.48)**	**<0.001**
Vitality	**0.52 (0.40, 0.66)**	**<0.001**	**0.33 (0.24, 0.47)**	**<0.001**
Locomotion	**0.26 (0.19, 0.38)**	**<0.001**	**0.26 (0.17, 0.40)**	**<0.001**
Psychology	**0.70 (0.47, 0.94)**	**0.022**	**0.39 (0.24, 0.62)**	**<0.001**
Vision	1.07 (0.65, 1.77)	0.789	**0.42 (0.23, 0.76)**	**0.004**
Hearing	1.06 (0.68, 1.66)	0.793	0.80 (0.47, 1.36)	0.408

^a^After adjusting for collection time, age, sex, smoking history, fear of falling, eating problem, CIRS-G score, polypharmacy, pain, and urinary incontinence. ^b^After adjusting for collection time, age, sex, marriage status, educational level, falling history, fear of falling, walking aids, eating problem, CIRS-G score, polypharmacy, and urinary incontinence. IC, intrinsic capacity; CIRS-G, Cumulative Illness Rating Scale for Geriatrics; OR, odds ratio; CI, confidence interval. Significance difference *P* < 0.05 was shown in bold.

## 4. Discussion

This study indicated that a higher IC composite score was associated with a decreased risk of falls and frailty in older hospitalized patients. Declines in cognition, vitality, locomotion, and psychology were the domains most susceptible to the impact of falls and frailty. Additionally, visual impairment was significantly associated with an increased risk of frailty.

This study found that IC impairments were common among older patients in hospital settings. Cognitive, sensory, and locomotor impairments were the three most prevalent domains of impairment, with percentage rates of more than 50%. The reported rates were higher than those in the Chinese community (44, 45) and inpatients with relatively healthy status at Xuanwu Hospital (25), but similar to those reported in Beijing Hospital (24), which may be attributed to advanced age, complex multimorbidity, and multiple functional loss due to hospitalization.

Studies have shown that falls are influenced by multiple factors in older individuals, among which functional capacity impairment plays a critical role in preventing the occurrence of falls (27, 46). Consistent with this study, we identified that higher IC levels, especially those related to the preservation of cognitive, vitality, locomotion, and psychological independence, were associated with a lower risk of falls. Liu et al. demonstrated that impaired vitality, locomotion, and psychology domains predicted the incidence of future falls within two years and that cognitive decline was associated with activities of daily living dependence in the future (21). Another study suggested that older adults with falls or those at risk of falls had at least three domains of IC decline (47). Thus, older inpatients with low IC composite scores and their domains constitute a high-risk group for falls in hospital settings. Fall prevention strategies in hospital settings should address IC assessment and management upon admission. The effect of low IC levels on fall risk may be explained by both direct and indirect mechanisms. First, impaired cognitive functions such as executive function, orientation, and memory dysfunction may influence the sensory information related to maintaining balance while walking and the ability to perceive and judge falls risk in older adults (48, 49). Second, poor nutritional status, especially inadequate protein intake and vitamin D deficiency reduces muscle mass and function and leads to the development of sarcopenia (50–52). These negative conditions may lead to a slow gait, poor balance, and immobility, thereby increasing the risk of recurrent falls (50, 53). Third, various depressive symptoms, including a negative self-evaluation, cognitive changes, poor sleep quality, and decreased physical activity, may cause fear of falling and falls (54–57). Fourth, evidence showed that sensory impairments were predictors of an elevated risk of falls (58, 59). Despite no significant difference between sensory impairments and falls risk in this study, it may be attributed to a high incidence of sensory impairments in older adults across fall risk groups. In addition, interactions between various impaired IC domains and multiple comorbidities may contribute to functional deterioration and fall occurrence.

With regard to the association of IC and frailty, our study documented that a higher IC composite score was associated with decreased risks of frailty. Older inpatients with IC domain impairments, except hearing loss, were found to be more prone to frailty. This is consistent with recent studies arguing that IC impairments are associated with frailty (60–62). A longitudinal study of the Multidomain Alzheimer’s Preventive Trial confirmed that each additional IC impairment was associated with a 47% increase in the risk of incident frailty (61). Mobility limitation, depressive symptoms, and vision impairment were associated with incident frailty at the five-year follow-up in community-dwelling older adults, but no significant associations between cognitive decline, malnutrition, or hearing impairment with frailty were observed (61). Another study conducted in China found that IC was significantly associated with incident frailty, and individuals with impaired vitality and locomotion domains were more likely to be frail compared with other domain combinations (62). In addition, IC impairment and frailty were regarded as overlapping and coexisting and had a synergistic effect on adverse outcomes. Liu et al. found that newly impaired vitality and locomotion domains were related to transitions from non-frail to frail status (63). Data from a study conducted in Singapore revealed that older adults with low IC who were prefrail/frail were more prone to exhibit poorer outcomes, including a higher proportion of transitioning to frailty or remaining frail, decreased activity of daily life function, and quality-of-life at one year when compared with older adults with high IC and robust or intermediate IC and prefrail (64). The 12-week Vivifrail multicomponent exercise program has been found to be an effective strategy to enhance IC, especially in terms of the locomotion, cognition, and vitality IC domains, in community-dwelling older adults with pre-frailty/frailty and mild cognitive impairment/mild dementia, compared to usual care (65). However, the above-mentioned evidence was mostly from the community rather than hospital settings. Further evidence from a larger sample, based on hospital settings, is warranted. Therefore, IC may better characterize frailty in older individuals in the hospital setting, and targeting a high-risk group based on low IC composite scores and its domain scores may provide additional intervention recommendations that go beyond current nutrition and exercise recommendations for physical frailty and help to optimize individualized care plans during hospitalization.

The results highlighted the negative associations of IC, fall risk, and frailty in older inpatients. In addition, the current study included a well-characterized older inpatient population and comprehensive assessment information from the CGA database of the geriatric department in our tertiary hospital. Nevertheless, this study has several limitations. First, because IC is a dynamic but reversible construct (66), regular monitoring of IC on admission and during hospital stays may contribute to detecting potential IC declines ahead of clinical adverse events, such as falls and frailty. However, the cross-sectional design hinders the possibility of examining the change in IC composite scores and negative events during the hospital stay. Further longitudinal studies to confirm causality are warranted. Second, convenience sampling strategies for hospital admissions and significant differences were observed in some characteristics between the included patients and the excluded patients, such as age and medication usage may have led to an inevitable selection bias. Therefore, these results should be generalized with caution. Finally, some domains were collected using subjective measurements, which may have introduced a subjective bias.

## 5. Conclusion

This study indicated that a decline in IC was associated with fall risk and frailty in older inpatients. Further prospective studies are needed to explore the longitudinal associations of in-hospital baseline IC and subsequent risk of falls and frailty.

## Data availability statement

The raw data supporting the conclusions of this article will be made available by the authors, without undue reservation.

## Ethics statement

Ethical approval was obtained from the Medical Ethics Committee of Zhejiang Hospital (2013-25). The studies were conducted in accordance with the local legislation and institutional requirements. The participants provided their written informed consent to participate in this study.

## Author contributions

SS, YX, and XC contributed to conceptualization and methodology. SS and YX analyzed the data and wrote the original draft. XZ, YY, and XW contributed to data collection. LC and HG participated in functional assessment. All authors contributed to implementing and revising the manuscript.
